# Nursing Care Coordination in Primary Healthcare for Patients with Complex Needs: A Comparative Case Study

**DOI:** 10.5334/ijic.6729

**Published:** 2023-02-02

**Authors:** Marlène Karam, Maud-Christine Chouinard, Yves Couturier, Isabelle Vedel, Catherine Hudon

**Affiliations:** 1 Faculty of Nursing, Université de Montréal, Quebec, Canada; 2 Department of Social Work, Université de Sherbrooke, Quebec, Canada; 3 Department of Family Medicine, McGill University, Quebec, Canada; 4 Department of Family Medicine and Emergency Medicine, Université de Sherbrooke, Quebec, Canada

**Keywords:** integrated care, care coordination, complex needs, nurses, continuing education, case study, soins intégrés, coordination des soins, besoins complexes, infirmières, formation continue, étude de cas

## Abstract

**Introduction:**

Despite nurses’ substantial role in care coordination, few education programs exist to better support them in this role. Identification of a set of core care coordination activities across heterogeneous care coordination programs would facilitate the development of a standard of practice. We sought to examine care coordination activities across two care coordination programs in Family Medicine Groups in Quebec, and their relationship to the program design.

**Methods:**

We performed a comparative case study of two care coordination programs in primary care targeting frequent users of healthcare services and people with Alzheimer’s disease and related disorders. Data collection included documents and semi-structured interviews with key informants.

**Results:**

Several activities were common to both programs, such as patient identification; assessment, development of an individualized service plan; and linking patients and caregivers with professionals and services. However, their components were different due to the impact of the integrated care program design, policy environment, and the target patient populations’ complex needs.

**Discussion:**

The homogeneity or heterogeneity of patients’ complex needs shapes their care trajectory and the intensity of their care coordination needs. As the complexity of these needs grows, so does the necessity to build the care coordinators’ capacity for integrated care.

## Abstract (fr)

**Introduction:**

Malgré le rôle important des infirmières dans la coordination des soins et des services, peu de programmes de formation existent pour mieux les soutenir dans l’exercice de celui-ci. L’identification d’un tronc commun d’activités de coordination des soins et services effectuées par les infirmières à travers différents programmes de coordination faciliterait l’élaboration d’une norme de pratique en vue de rehausser leur formation à cet égard. Cette étude examine les activités de coordination des soins et services effectuées par les infirmières dans deux programmes de coordination hétérogènes déployés dans les groupes de médecine de famille au Québec, et la façon dont la conception du programme impacte celles-ci.

**Méthodes:**

Nous avons réalisé une étude de cas comparative de deux programmes de coordination des soins et services en première ligne ciblant les usagers fréquents des services de santé et les personnes atteintes de la maladie d’Alzheimer et des troubles apparentés. La collecte de données comprenait une analyse documentaire et des entretiens semi-dirigés avec des informateurs clés.

**Résultats:**

Plusieurs activités étaient communes aux deux programmes, telles que l’identification des patients ; évaluation, élaboration d’un plan de services individualisé ; et la mise en relation des patients et des soignants avec des professionnels et des services. Cependant, leurs composantes étaient différentes en raison de l’impact de la conception du programme de soins intégrés, de l’environnement politique et des besoins complexes des populations de patients cibles.

**Discussion:**

L’homogénéité ou l’hétérogénéité des besoins complexes des patients façonne leur trajectoire de soins et l’intensité de leurs besoins de coordination des soins. À mesure que la complexité de ces besoins augmente, la nécessité de renforcer la capacité des coordonnateurs de soins en matière de soins intégrés augmente également.

## Introduction

Ensuring coordinated care between health and social care services has become a well-established policy and practice concern in most developed countries [1]. Indeed, care coordination has been identified as a key strategy that has the potential to improve the effectiveness, safety, and efficiency of healthcare systems [2]. However, and despite the proven benefits, care coordination is still difficult to accomplish. Barriers include the high level of health system fragmentation [3,4], the growing complexity of patients’ health and social care needs [5,6], the numerous participants and organizations involved in coordinating care [7], the shared accountability of the process, which contributes to ambiguity as to who is responsible for making it run smoothly [8], the underfunding and lack of reimbursement of added time and effort required for care coordination [6,8], and the various health information technology challenges [9]. Consequently, improving care coordination has required effort across all levels, i.e. patient and provider, services and organizations, and health system [8].

In the province of Quebec (Canada), and in other international health systems, care coordination in primary healthcare for patients with complex health and social needs poses a significant challenge. In 2000, Family Medicine Groups (FMGs) were implemented to improve the organization and delivery of primary healthcare services. FMGs are primary healthcare practices that consist of a group of family physicians working closely with registered nurses and other health and social care professionals to provide services to registered patients [9].

In recent years, several projects have been carried out in FMGs to reduce fragmentation and improve healthcare delivery through better coordination of services for patients with complex needs. These projects targeted patients with Alzheimer’s disease and related disorders [10], cancer survivors [11], and frequent users of healthcare services [5], among others. Within these projects, registered nurses often played the role of care coordinators for the different target patient populations. Although this role can be undertaken by professionals from various backgrounds [12], for these projects, nurses were deemed a relevant choice based on the population of interest’s needs that require nursing competencies, and contextual factors such as the availability of nursing staff in every FMG.

The paradox is that while nurses are increasingly expected to play a substantial role in care coordination, they still know little about care coordination principles, namely because teaching these principles is still not fully integrated in the primary care nursing curriculum or in continuing education programs [13]. Enabling healthcare professionals to work in interdisciplinary teams, to collaborate, communicate and integrate care as part of teams is thought to be a major lever for the provision of high quality and coordinated care [14]. A recent study showed that nurses’ activities in FMGs do not always include a high level of collaboration—such as face-to-face meetings, individualized service planning, or problem resolution—between the members of an interprofessional team, let alone with the services and professionals of regional health authorities [15].

Another challenge is related to the fact that care coordination interventions vary widely in structure [16]. In Quebec, care coordination projects presented significant structural variations across FMGs. Moreover, when several care coordination projects were conducted in one FMG, they included a different list of care coordination components, carried out by the same nurses. Furthermore, within the same FMG, one project may be operationalized differently, according to the nursing staff’s work habits and the family physician’s preferences.

There is a need to identify a set of “core activities” that would be relevant to all patients with complex needs across heterogeneous care coordination programs if the aim is to develop a standard of practice for nurses performing this task.

In this study, we aim to examine the differences and similarities in terms of care coordination activities across two heterogeneous care coordination programs in FMGs in Quebec, and how these similarities and differences are related to the program design.

## Conceptual frameworks

For the description of the care coordination program design and policy environment, we used the Integrated Care Case Study Descriptive Template. This framework was developed to enable the comparison of integrated models of care across diverse geographies and contexts and has been validated through multiple research projects. The template examines design elements and activities of the integrated care program, and the policy context that supported the program [17].

As for the care coordination activities within the intervention, in a recent scoping review, the authors of this study developed a model of nursing care coordination activities for patients with complex needs [18]. In this model, activities were grouped into three categories, namely the activities that target the patient, family and caregivers; those that target health and social care professionals and services; and those that bring the patient and professionals together. A fourth category was identified as cross-cutting, supporting and enhancing every other activity, i.e. interpersonal communication and information transfer. We used this model to examine the nursing care coordination function in the current study.

## Methods

### Design

We followed a case study approach to compare the nursing care coordination function in two programs in Quebec. Merriam (2009) defines case study methodology as “an in-depth description and analysis of a bounded system” [19, p. 40].

The case is “a single entity, a unit [that you] can ‘fence in’ [like] a single person who is a case example of some phenomenon, a program, a group, an institution, a community, or a specific policy” [19, p. 40]. In our study, the case was defined as the care coordination program in FMGs. A deep understanding of the program as a whole seemed like an essential prerequisite for capturing the essence of the nursing function within this program.

### Case selection

For Merriam (2009), cases are selected based on the research purpose and question, and for what they could reveal about the phenomenon or topic of interest [19, p. 46]. Thus, we sought to include two programs that were implemented in FMGs, for patients with complex health and social care needs, and where care coordination is performed by nurses, in order to identify the common core of these two programs as well as the cross-cutting nursing skills, which are essential to optimal care coordination. Also, the programs were purposely selected to provide variation in target patient populations to help us investigate how different patients’ needs might affect care coordination. Therefore, Case 1 was “the V1SAGES program” which is a case management program, which aimed to improve care for frequent users of healthcare services [5]; and Case 2 was the “Quebec Alzheimer Plan”, which aimed to improve the care provided to people with Alzheimer’s disease and related disorders [10].

### Data collection

Data collection took place between August 2020 and March 2021. Two types of data were used, documents and semi-structured interviews with key informants. These informants are either project managers or senior researchers who had an in-depth knowledge of the program, and were involved in the development, implementation and evaluation of the two programs. Together, these methods provided a synergistic and comprehensive view of the care coordination programs being studied. We looked for research publications, unpublished records such as master’s theses, publicly available records, and private documents (such as protocols and standards of care, care coordinators’ training records, clinical tools, communication and coordination tools, and patients’ informed consent forms) (see Appendix A). Five semi-directed interviews were also conducted with key informants and lasted approximately 90 minutes each. The interview guide was based on the dimensions of the Integrated Care Case Study Descriptive Template. Interviews were instrumental in refining and completing the preliminary results of the documentary analysis in an iterative approach.

### Data extraction and analysis

For each program, the following variables were extracted from the documents and completed through semi-structured interviews: policy context, history, detailed description and implementation phases, health and social care partners and community resources involved, characteristics of target patient populations, care coordination activities, clinical tools, coordination tools, staffing model, human and material resources, and evaluation.

Data collection (documents and interviews) and analysis were performed concurrently. NVivo 12 software was used for data organization and management. Analysis was thematic [20], using both deductive (using the Integrated Care Case Study Descriptive Template and the model of nursing care coordination activities for patients with complex needs) and inductive approaches, and followed a two-stage process. First, a within-case analysis studied each intervention comprehensively and provided an in-depth description of the above-mentioned variables. Then, at the end of this stage, a comparative cross-case analysis was performed. It aimed to develop a global explanation and *“to build abstractions across cases*” [19]. In concrete terms, cross-case analysis aimed to identify the common core of the two programs (regardless of the specificity of the target population), the differences linked to the target population, and the cross-cutting nursing care coordination activities.

## Ethical considerations

This study received institutional approval from the Comité d’Éthique de la Recherche du Centre Intégré Universitaire de Santé et de Services Sociaux de l’Estrie – Centre Hospitalier Universitaire de Sherbrooke.

## Results

### Case 1: The V1SAGES program

#### Description of the project

The V1SAGES (Vulnerable Patients in Primary Care: Nurse Case Management and Self-Management Support) program was initiated in 2012, in the Saguenay–Lac-Saint-Jean (SLSJ) region of Quebec. The evidence indicated that, despite what the creation of FMGs has offered in terms of improved accessibility and continuity of care for certain patients with chronic diseases [21], the most vulnerable groups still face major challenges, mainly due to the lack of service coordination and integration [22]. These vulnerable patients often attempt to fill the existing gap between their complex needs and the ability of the healthcare system to meet their needs by using health and social care services excessively and in an uncoordinated way [23]. The development of case management (CM) programs for frequent users was identified as a potential solution following a major consultation process organized by the *Agence de santé et des services sociaux du Saguenay–Lac-Saint-Jean* [24]. Stakeholders involved in this consultation included health and social care professionals, community-based organizations, managers, policymakers, and researchers. This decision was reinforced by two main criteria: A significant body of evidence in the literature on the effectiveness of CM on emergency department use and cost as well as on social and clinical outcomes [25,26,27]; and, a recent promising—though still in the early stages of development—experience of CM programs in the six health and social services centres (HSSC) of the SLSJ region. HSSCs include hospital, community, and long-term services, but not FMGs. The expansion of CM within FMGs was thought to be a complementary intervention that would help to better respond to the complex needs of vulnerable patients and improve service integration [22].

Pre-implementation interviews conducted with stakeholders aimed to describe the contextual factors, the existing services and their integration level, as well as the challenges and issues related to the implementation of CM intervention [22]. The intervention was then rolled out in four FMGs over a period of six months, and had four components: “Evaluation of patient needs and resources, implementation and sustainability of an individualized service plan tailored to patients’ priorities with the collaboration of the healthcare and community partners, care coordination among the healthcare and community partners, and provision of self-management support for patients and their families” [5, p. 233].

Registered nurses (RNs) were assigned as case managers. The majority were already working within the FMGs and a few were recruited to build nursing staff capacity for this intervention. They had a bachelor’s degree and 10 to 17 years of experience [28]. Their expertise and existing relationships with the medical teams and partners (e.g. community pharmacists) were considered valuable assets for this intervention [22]. Nurses received training to fulfill this role. Nurse case managers spent approximately half of their time in CM, with a caseload of approximately 25 frequent users of healthcare services [28]. During this phase, the implementation-related challenges and obstacles faced by FMG teams and stakeholders were identified.

As one key informant explained, doctors played a central role in facilitating or obstructing the implantation:

“In one of the FMGs, the medical manager said, ‘No, my nurse will not play the role [of case manager]’; so even if the nurse shows a lot of leadership, is very skilled, unfortunately, it [the intervention] stopped there for this clinic”.

Another informant confirmed by saying:

“You definitely need a change champion, but it still takes a lot of change facilitation for it to happen; a lot of collaboration with managers, regional authorities and FMGs”.

The next phase—post implementation—analyzed the implementation process. Studies also assessed the effect of the V1SAGES intervention on psychological distress and patient activation, and stakeholders’ perception of the intervention [5] as well as the experience of patients and their family members with the CM intervention [28].

In 2018, a 4-year project was launched with the objective of implementing this same CM intervention in ten primary care clinics across five Canadian provinces (Newfoundland, Nova Scotia, New Brunswick, Quebec, and Saskatchewan). As one key informant explained during the interview:

“We [the research team] built on our recent experience with V1SAGES and the lessons we’ve learned to develop an improved CM intervention on a larger scale. We called the enhanced CM intervention PriCARE (Partners for Patients First)”.

Table 1 presents the common design and policy environment of V1SAGES and PriCARE as well as the differences between the two CM interventions. Ongoing studies within PriCARE aim to identify the next steps to expanding this CM intervention in primary care across Canada. Thus, they examined the implementation of the intervention, contextual factors, mechanisms, and barriers and facilitators. Research methodologies include an implementation analysis, a realist evaluation and a logic analysis of the intervention [23,29,30,31,32]. They also analyze patient engagement across the different phases of this study [31].

**Table 1:** V1SAGES and PriCARE: Design and policy environment.


	SEGMENTATION	COORDINATION	ENGAGEMENT	SUCCESS MEASURES	POLICY-RELATED CONTEXT

Description	Defining and applying rules to identify and recruit patients who are likely to benefit	An intake process to characterize needs, mechanisms for coordination across institutions and sectors such as health and social care	Support for shared decision-making, self-management and support for caregivers	How programs defined success, their level of maturity and any evaluation work conducted	Policy innovation to support integrated health and social care, and innovation in care delivery

	**Target group:** frequent users of healthcare services with chronic diseases	**Intake:** Intake depends on the eligibility criteria. Then an initial assessment is conducted by the CM using a standard tool filled out during in-person consultation (home visit or consultation in the FMG). It includes a comprehensive history and assessment of health and social care needs	**Patient engagement:** Explicit culture of shared decision-making embedded in professional training and approach. Activities that support patient engagement include goal-setting with patients, developing individualized care plans, and patient partner involvement in the different phases of research	**Maturity level:** V1SAGES: Began in 2012 and the intervention is mature in terms of design, development and reflection on the related issues, yet the scale-up is still in its early stages PriCARE is indeed still at its early stages. The expansion targets the same population although eligibility criteria are slightly different	**Financing:** V1SAGES: At the initial set-up phase, the funding to carry out the CM intervention (mainly RNs’ salaries) came from research funds, then the FMGs took over with their operational funds. PriCARE: Nurses are paid through the primary care clinics’ operational funds

	**Entry point:** V1SAGES: A list of frequent users is generated by the regional health authority information system and submitted to the FMG professionals’ clinical judgement. Thirty patients per FMG are included. PriCARE: Each FMG identifies 30 patients with the most complex needs by searching administrative data and clinical records in addition to using their clinical judgement. Future orientation: A case-finding tool was developed. It enables early identification, in the ED, of patients with complex needs at risk of high use of healthcare services. Patients identified early would be referred to a CM program [33].	**Primary care providers:** All patients have access to their GP who works closely with the nurse CM (who is responsible for coordinating care and services for them). Other health and social care providers are called upon to intervene when necessary.	**Patient self-management:** Self-management support for the patient and family is a main component of the intervention and CMs are trained in motivational interviewing and other methods aimed at promoting patient self-efficacy and empowerment. CMs are attentive to family and caregiver needs and provide support when possible. Nursing interventions with the patient’s family and caregivers are integrated in the primary care nursing curriculum.	**Measures (current goals):** The program goals are aligned with the Quadruple Aim: better health outcomes, better patient/caregiver experience, better provider experience, and lower costs.	**Staffing:** The CM intervention is innovative in that it redefines and creates new roles and responsibilities for nursing staff. Nurse CMs are employed by FMGs which promotes the sustainability of the intervention in the participating FMGs. V1SAGES: Nurses spend half of their time in CM, and the remaining half in other primary care duties. PriCARE: Nurses dedicate one day per week to CM activities.

	**Eligibility:** V1SAGES: Patients with 3 or more ED visits, hospitalizations, or both, in the previous 12 months. Patients aged 18 to 80 years who have at least one chronic disease. Patients with serious cognitive problems were excluded. PriCARE: Patients living with at least one chronic condition; who are frequent ED users (as defined by ≥4 ED visits or hospitalizations in the previous year); and a score ≥17 on the INTERMED-Self-Assessment Questionnaire evaluating complex healthcare needs. Development of eligibility criteria was driven by quality issues.	**Integration:** Involves a wide range of health and social care services, community organizations, and specialized clinics and services, depending on each patient’s complex needs. The CM communicates directly with the targeted professionals. Meetings (virtual or in-person) are scheduled to establish and review the ISP with the patient.	**Caregiver engagement:** This is a clear component of the intervention. With the patient’s consent, the caregiver is involved in the development of the ISP. The caregiver may stand in for the patient at any point during the intervention.	**Data collection:** No routine data are collected; however, many studies were conducted or are in progress.	**Governance:** V1SAGES: A CM committee was formed with the aim of linking CMs working in hospitals with those working in FMGs. The committee is officially part of the governance structure of the IUHSSC. Also, FMG and IUHSSC managers meet regularly to review CM performance and other issues. CM communities of practice (mentored by an expert) were also formed to share knowledge, discuss potential difficulties and proceed with necessary adaptations or changes to the intervention. PriCARE: Patient and family partner communities of practice were formed in addition to those of the CMs. Stakeholders who collaborate with the academic research team include a patient partner, who plays a central role as an “external change agent”, the two types of communities of practice, managers and GPs, and decision-makers. Each stakeholder is involved according to their interest, availability and expertise.

		**Transitions:** Other than the ISP, there are no structured protocols for care transitions across sectors or care settings. The CM facilitates patient transition through direct communication with services.		**Evaluation:** Several formal research studies were conducted to evaluate the intervention, its implementation and outcomes (see description of the program).	**Health and social care data sharing structure:** There is no common data sharing structure. Innovation is related to the ISP, which constitutes the main communication tool between providers, and to the role of CMs in establishing communication channels with providers and organizations, including a communication plan for emergencies.

		**Information sharing:** Only co-located health and social care professionals share an EHR. There is no operational common data platform for sharing data across providers and settings. Information transfer occurs through electronic and paper referrals.			**Care delivery innovation:** To our knowledge, it is the only intervention in Quebec developing a CM role for primary care nurses. Patients are involved in every decision throughout the intervention. PriCARE: Patients are also engaged in every phase of the research.


Legend: CM: Case manager; ED: Emergency department; EHR: Electronic health record; GP: General practitioner also known as family physician; ISP: Individualized service plan; IT: Information technology; IUHSSC: Integrated university health and social services centres (resulted from merging neighbouring health and social services centres with all the other public healthcare organizations, such as youth centres, rehabilitation centres and university teaching hospitals under a single governing body per regional territory); MD: Medical doctor; RN: Registered nurses; SW: Social worker.

### Case 2: The Quebec Alzheimer Plan

#### Description of the plan

Alzheimer’s disease (AD) and other related major neurodegenerative diseases (MND) have a considerable impact on Quebec society, at all levels, and this impact is worsening dramatically due to population aging [34]. Among Quebeckers aged 65 and over, 100,000 had the disease in 2009, and this figure is expected to rise to 200,000 by 2030 [10]. Moreover, up to 80% of people with AD have at least one other chronic disease [35]. In addition to causing disability among older people, AD affects families and informal caregivers’ health and financial status, and puts tremendous pressure on the health system and on society [10,34,36]. For all these reasons, MND care became a priority for the Quebec Ministry of Health and Social Services (MSSS), which mandated a committee of experts to develop an action plan on AD and related disorders, in 2007. Two years later, the committee delivered its report in which it presented seven priority actions, with 24 recommendations on how to carry them out, and a strategy for implementing the plan with five related recommendations [10]. Following the publication of the report, several FMGs set up spontaneous interventions in order to improve the identification, diagnosis and care of people with AD at the primary care level.

The MSSS commissioned a research team to provide an accurate picture of these interventions in order to effectively implement and operationalize the recommendations included in the expert committee report [37]. Then, an action plan was developed based on some of the recommendations from the experts’ report. Indeed, although the experts’ recommendations cover the continuum of care, the Ministry team had to prioritize the measures to be implemented, as they had a limited annual budget allocation. Therefore, and considering the growing consensus among Canadian experts and decision-makers that the focus of dementia care should shift from specialist to primary healthcare [38], strategy implementation focused on strengthening the primary care level to ensure early diagnosis of and follow-up with patients with AD and their caregivers, in the community. Although there is still no treatment to cure AD, the benefits of early diagnosis (such as rapid access to medication, the ability to plan for the future, and the ability to postpone institutionalization) are well established [39]; and, primary healthcare clinicians are ideally positioned to establish this early diagnosis and ensure follow-up [38].

One of the priorities established in the report concerned “providing access to personalized, coordinated assessment and treatment services for people with Alzheimer’s and their family/informal caregivers” [10, p. 16]. To this end, the report recommended the implementation of a physician-and-nurse partnership, originating in FMGs and including the patient and caregivers, in which nurses would play the role of Alzheimer’s patient navigators and be in charge of care coordination and continuity of patient services. During one interview, a key informant stated:

“The key was, the real key, the real concrete product, was to create a nurse-doctor duo focused on assessment, hoping that the joint assessment would have a culture-changing effect. And that, I think, was pretty good. I think the plan [the Quebec Alzheimer Plan] can be quite proud of the culture shift effect around the time of the evaluation”.

Other recommendations related to this specific priority covered the support needed by FMGs to reach their objectives in terms of staffing, training, standardized assessment tools and practice guides, and specialized teams and services.

Implementation of the MSSS plan (the Quebec Alzheimer Plan [QAP]) was rolled out in two phases: a pilot phase and a deployment phase.

The pilot phase (2012–2016): The MSSS launched a call for proposals for the development of innovative, local projects tailored to each FMG’s needs and context. Therefore, local actors had room to maneuver as to the type of proposals they designed as long as they targeted the Ministry’s objectives: Building the FMG’s capacity to diagnose AD early and follow-up with patients and caregivers in the community, through an interdisciplinary approach where FMG registered nurses are the care coordinators, acting as patient navigators [40,41].

At least one nurse per project was added to the team. All nurses received training focused primarily on the interdisciplinary approach in FMGs to identify, assess and follow up with patients and caregivers [42]. They kept their initial caseload of patients, and no specific specialization was required from nurses since these responsibilities were rather consistent with the role of FMG registered nurses, as stipulated in a consultation document between the Quebec Order of Nurses and Quebec Federation of General Practitioners [43].

“The idea was that every nurse could manage this [new responsibility], because the QAP did not want a patient who has dementia and diabetes and hypertension to see one FMG nurse for their diabetes, another nurse for their dementia, and another nurse for their hypertension, thus recreating a fragmentation that is completely deleterious to the patient”.

Nineteen projects, led by family physician “advocates”, were retained (a total of 42 FMGs), and received protocols, training and funding as well as the support of designated project managers for two years (2014–2016) [44,45,46].

This phase was characterized by a great diversity of projects and enabled the experimentation of a variety of changes in clinical practice, the development of assessment tools and clinical pathway guides, and the identification of best practices [47]. In fact, a continuous evaluative study was set up during this phase to enable monitoring and progressive adjustment of service structures based on encountered challenges and to support their transferability to other contexts [48], in other words, to prepare for the scale-up phase. For instance, the research team explored the conditions associated with the change in FMGs following the passive dissemination of recommendations for the diagnosis and management of AD [49]. They also assessed the impact of the QAP on the detection and management of AD and other neurocognitive disorders in primary healthcare [50]. A questionnaire evaluating the knowledge, attitude and practice of family physicians regarding both dementia care and the QAP was also developed and validated in order to measure progress in the implementation of dementia strategies and their impact [38]. Our key informants believed that:

“many family physicians were not initially convinced that dementia is a health problem that should be managed at the primary healthcare level. It took a lot of energy and resources to convince them. I think the added value of this project was also that those doctors now know that. The relationship between the FMGs and the memory clinic is clearer: Everyone has a better understanding of what they have to do”.

The deployment phase (2016–2020): The scale-up of the QAP to the entire province of Quebec. Under the responsibility of the regional health authorities, the implementation of the best practices identified in the previous phase began in FMGs and then expanded to all primary care services offered to patients with AD and their caregivers. The MSSS maintained the funding and the support of project managers for another three years and published an “Implementation guide for the deployment of the best clinical and organizational practices” [51]. The guide provided healthcare professionals with an interdisciplinary clinical process to be followed at the primary care level and helped clarify the referral process to memory clinics. It also enabled the recognition of the nurses’ role in the detection and diagnosis of AD and related disorders. Beyond 2021 (Phase 3), the aim is to gradually integrate the management of dementia into a comprehensive care approach for older people at the primary care level and across care levels with a focus on teamwork in FMGs and on interorganizational collaboration throughout the continuum of care. Table 2 presents the design and policy environment of the QAP.

**Table 2:** Quebec Alzheimer Plan: Design and policy environment.


	SEGMENTATION	COORDINATION	ENGAGEMENT	SUCCESS MEASURES	POLICY-RELATED CONTEXT

Description	Defining and applying rules to identify and recruit patients who are likely to benefit	An intake process to characterize needs, mechanisms for coordination across institutions and sectors such as health and social care	Support for shared decision-making, self-management and support for caregivers	How programs defined success, their level of maturity and any evaluation work conducted	Policy innovation to support integrated health and social care, and innovation in care delivery

	**Target group:** Patients with major neurocognitive disorders (Alzheimer’s disease and other NCD) and their caregivers.	**Intake:** In case of clinical suspicion, a comprehensive assessment of mental and cognitive states and depression is conducted by the GP-RN duo, using validated tools such as MMSE, MoCA and GDS-15 or GDS-5. RNs also assess the patient’s functional ability (ADL, IADL) and their health and social care needs. A diagnosis is made based on the disease history, cognitive and functional decline results.	**Patient engagement:** The QAP promotes the autonomy, independence and participation of patients and caregivers. In the clinical context, patients’ priorities are assessed and their choices are taken into consideration for the decision-making process.	**Maturity:** The QAP is currently well established and mature after its second implementation phase. This phase was rather a progressive development of best practices, on a provincial level, through access to an implementation guide, training and a change management strategy.	**Financing for model:** Public funding. For the pilot phase (2014–2016), an annual budget of five million dollars was allocated by the MSSS to implement the QAP in 19 selected projects. Each project received $250,000 per year (over 2 years). A recurring budget ($225,000 per regional health authority) was allocated afterwards to support the deployment of best clinical and organizational practices in all regional health authorities throughout Phase 2. Sustainability of the QAP is ensured through recurrent funding (dedicated to staff professional development) and the allocation of regional nurses mandated to train staff and update training content. It should be noted that the allocated amount has remained the same but is now spread over many more FMGs.

	**Entry points:** Self-referral (patients and families) or by professionals who suspect a neurocognitive disorder. Although systematic screening is not recommended, a vigilance zone is applied for those at high risk (65 years old and above with risk factors; 75 years old and above who renew their driving licence).	**Primary care providers:** A GP-nurse partnership with the patient and their caregiver is established. These two primary care providers are responsible for early diagnosis, treatment (if needed), management and referral of patients to relevant providers and services, especially referral to cognition clinics for complex cases. Other care providers (social workers, occupational therapists, pharmacists, special education technician) were added to the existing teams and located either in primary or specialized care or shared by the two levels of care. Unfortunately, little effort was put into coordination with regional health authority programs aimed at supporting the autonomy of the older people.	**Patient self-management:** This is sometimes difficult to operationalize given their functional and cognitive status. Patient education, support and counselling are provided by nurses during regular follow-up visits.	**Measures (current goals):** Although the plan’s recommendations cover the continuum of care, the implementation strategy promotes building the capacity of FMGs to ensure the early diagnosis and overall monitoring of people with Alzheimer’s and their caregivers in the community.	**Staffing model:** The pilot projects resulted in the addition of at least one nurse per project, eight social workers, a few occupational therapists, a pharmacist and a special education technician. These professional resources were added either to the IHSSC, to the FMGs, or shared between the FMGs and the specialized organizations. Only a few were assigned to specialized organizations. Part of the budget of the deployment phase was used to train and provide equipment to healthcare providers. The RNs kept their initial caseload of patients and were trained to pay particular attention to patients who present signs of AD or were at high risk.

		**Integration:** Available personalized and coordinated **s**ervices include (to varying degrees) primary care in the community, psychosocial resources such as Alzheimer Society support centres, home care programs, specialized care such as specialized memory clinics and specialized teams dedicated to behavioural and psychological symptoms of dementia.	**Caregiver engagement:** This is a major component of the QAP as caregivers are considered valuable partners. For instance, they are actively involved in monitoring changes in the patient’s cognitive and functional status. Caregivers are also entitled to psychoeducational and support services to help them cope with the patient’s condition, better navigate the health system and the community network, and establish an end-of-life plan. When deemed necessary, RNs proceed with an assessment of the caregiver’s burden. Patients and caregivers were not involved in the co-design of the first phase of the QAP. However, Phase 2 involved patients’ and caregivers’ representatives (Alzheimer Society) in the development plan.	**Data collection:** This was done to varying degrees in the FMGs. The recommended indicators were difficult to collect or perceived as irrelevant by the FMGs. They prioritized staff training in assessment and identification of patients with AD, and teamwork. Therefore, they collected process indicators on these two variables (i.e number of staff trained). There were also difficulties with collecting harmonized data across all FMGs. Finally, data collection was assigned to RNs. Their overloaded schedule and turnover impeded the process.	**Governance structure:** Shared governance model combining top-down components and a bottom-up approach. National priorities were established by the MSSS (who set up a committee including partners and researchers), and professionals were asked to design their local projects according to their local realities while still aiming to achieve the objectives identified by the MSSS. Professionals received the support of designated project managers for coordination in terms of governance and change management.

		**Transitions:** The presence and use of a referral process and a structured protocol for care transitions across sectors or care settings varied between settings. Overall, RNs worked as patient navigators and advisors. They linked patients and their caregivers with home care, community care and specialized services but played a minimal role in optimizing transitions in the event of hospitalization.		**Evaluation:** A continuous evaluative study was set up during project implementation to enable monitoring and progressive adjustment of service structures based on the challenges posed by their implementation, and to support their transferability to other contexts. Evaluation of Phase 1 focused on the impact of the QAP on the quality of services provided by FMGs. Evaluation of Phase 2 focused on monitoring change management.	**Health and social care data sharing:** Although it was one of the recommendations, no innovative approach for sharing data across providers and settings was established. This was mainly due to the lack of interoperability with the existing EHR.

		**Information sharing:** A common EHR allows information sharing between healthcare professionals within an FMG. There is no operational common data platform for sharing data across providers and settings. Communication is considered suboptimal by providers. Channels mainly include referral letters and reports.			**Care delivery innovation:** The 19 pilot projects all presented innovations in the health and social services network, the main objectives of which are to diagnose AD and other major NCD more quickly in primary care, as well as to improve follow-up, in the community (as opposed to the specialized care level), with people with Alzheimer’s and their caregivers. Decentralization of clinical governance enabled the development of a broad diversity of innovative projects.


AD: Alzheimer’s disease; ADL: activities of daily living; IADL: instrumental activities of daily living; GP: General practitioner also known as family physician; MMSE: Mini Mental State Examination-Folstein; MoCA: Montreal Cognitive Assessment; GDS-15 and GDS-5: Geriatric Depression Scale; MSSS: Ministry of Health and Social Services. IHSSC: Integrated health and social services centres; IUHSSC: Integrated university health and social services centres; NCD: Neurocognitive disorders.

### Cross-case analysis

#### Nursing care coordination activities as related to the program’s design (segmentation, coordination, engagement, and success measures)

In both programs, it is well established that care coordinators perform their activities while applying the principles of interprofessional collaboration, patient and family-centred care and collaborative leadership. Care coordination activities undertaken by case managers and patient navigators are shown in Table 3 as related to the programs’ designs. However, it is important to remember that the QAP was implemented in all FMGs across the province with various degrees of implementation and that FMGs had considerable autonomy over the design and the implementation of their projects. Therefore, some care coordination activities may have varied from one FMG to another.

**Table 3:** Nursing care coordination activities across cases.


	V1SAGES AND PRICARE	QUEBEC ALZHEIMER PLAN

**Segmentation:** Care coordinators play an essential role in both programs for the identification of eligible patients with complex health and social care needs	**Identify patients who could benefit from the CM intervention:**	**Identify patients who could benefit from a cognitive evaluation:**

	Case managers: Use their clinical judgement to complement objective data from EHR or administrative databases related to frequent use of care services Use validated clinical assessment tools (the INTERMED-Self-Assessment Questionnaire) to assess complex health needs Consult patient EHR and clinical record from the hospital (if available) Understand the reason for frequent ED visits and for hospitalizations Identify the patient’s physical and/or mental illnesses Identify social challenges such as insecure housing or employment, poverty, violence, substance use disorders. Document the health and social services previously provided to the patient, as well as the names, roles and contact information of professionals currently involved with the patient or who may eventually be called upon to participate in the care of the patient. Confirm whether the patient’s current situation requires a CM intervention (to be discussed with the family physician and key healthcare providers)	Patient navigators: Recognize a clinical situation or memory complaints reported by patients or caregivers that should trigger an evaluation Pay particular attention to high-risk patients Ask questions related to their memories or use the Mini-COG assessment tool (a 3-minute instrument that can increase detection of cognitive impairment in older adults). High-risk patients are: – aged 65 years old and over with one or more risk factors: CVA, TIC, recent delirium, old age depression, and Parkinson’s – aged 75 years old and over who need to renew their driving licence

**Coordination:**	**Assess comprehensive patient and family needs and goals:**	**Assess cognitive and functional status:**

	Assessment (and follow-up) could be performed during home visits if necessary. Case managers: Validate the information collected from the medical records with the patient Complete and specify the assessment that was initiated in the identification phase Assess and identify the patient’s personal needs, goals, priorities and preferences for future services and resources. Inform the patient that they have the right to appoint an advocate that may be someone other than a family member Involve the family and caregivers, with the patient’s consent Establish a final list of care professionals that will be invited to examine the patient’s situation (healthcare and social services professionals, managers or representatives of community organizations) Seek the patient’s consent to communicate with potential care professionals throughout the intervention Ensure that the patient understands and agrees to the creation of an individualized service plan	With the patient’s consent, an initial assessment is performed in the presence of a caregiver. Patient navigators: Assess previous medical history and family history, current social support, psychosocial context, etc. Evaluate the history of the memory complaint that triggered the visit Use validated assessment tools: – MMSE (Mini Mental State Examination-Folstein) – MoCA (Montreal Cognitive Assessment) – GDS-15 or GDS-5 (Geriatric Depression Scale) Assess the functional status, specifically the ADL and IADL Question caregivers about their perceptions of danger and the strategies to be implemented if they had to be away from home and leave the person alone Run blood tests or CT scans (if a collective prescription is used) Refer to the “*Clinical process aimed at treating behavioural and psychological symptoms of dementia*” in case of relevant symptoms Transfer information to the GP

	**Develop an individualized service plan (ISP):**	**Develop a nursing therapeutic plan (NTP):**

	Prepare the patient for an ISP meeting: Explain what an ISP meeting is, the role they will have to play and the importance of sharing their wishes and preferences during the meeting Seek the patient’s consent to invite relevant health and social care providers and caregivers to the meeting Use simple and clear language and be open to the patient’s views as a partner Plan the ISP meeting: Review the list of care professionals and relevant providers who will be invited to the ISP meeting Communicate directly with the targeted care professionals to request their involvement Ensure that the involvement of care professionals is clear Establish a detailed agenda for the meeting Communicate with the patient to reconfirm consent regarding the professionals who will participate in the meeting Lead the ISP meeting: Invite the care team to collaboratively examine the patient’s situation, needs and preferences prior to the patient’s arrival Develop the ISP with the patient and their advocate upon their arrival: Consider their needs and prioritize what they want to address Establish the preferred methods of communication and strategies for exchanging information with the group Write up the ISP in plain language Validate that the patient understands and agrees to it Transmit the ISP to the patient and care professionals	The nursing therapeutic plan (NTP) records the nursing plan and instructions for clinical monitoring and care, and reports on the evolution of the patient’s priority problems and needs. Patient navigators share their NTP with other healthcare professionals.

	**Follow-up:**	**Follow-up:**

	Establish with the patient their preferred method for reaching the case manager Follow up regularly with the patient’s primary care providers, ensuring active engagement Review the ISP at least once every 3 months Verify if the patient’s goals have been attained Reassess the situation with the patient and adjust the ISP as necessary (if the patient desires a change, or if a care professional identifies any issue) Monitor the current medications and changes (introduction of a new molecule, resuming medication, etc.)	Once the diagnosis has been established and announced by the GP (in the presence of the RN when possible), patient navigators: Re-explain the diagnosis to the patient and caregivers if needed Provide the patient and caregiver with their contact information and inform them of their availability Educate the patient and caregiver about the disease and actions to be put in place to manage the behavioural and psychological symptoms of dementia Discuss the diagnosis and treatment plan with the patient and caregivers Assess the patient and caregiver’s psychological state and needs Provide the patient and caregiver with information on the following: the need for a driving assessment medication risk assessment financial risk assessment
		Provide counselling on difficulties that arise in daily life as well as moments of discouragement with the person and their loved ones Provide counselling on medico-legal aspects Use collective prescriptions to monitor medications Write and update a therapeutic nursing plan to ensure personalized clinical follow-up Two weeks later (then four to six weeks later): Verify patient and caregiver’s understanding of the situation, the situation itself and the treatment plan In case of medication intake: check side effects, adherence, tolerance, weaning if needed Six months later: Repeat the cognitive and functional evaluation Reassess the patient’s and caregiver’s psychological state and needs Reassess side effects, adherence, tolerance to medications, and manage medications following a collective prescription Reassess the need for home care services Refer the patient and caregivers to relevant resources if needed Ask about the patient’s driving ability and inform the GP Schedule a follow-up in six months

	**Coordinate care and services:**	**Coordinate care and services:**

	In addition to activities included in the planning, development and follow-up of the ISP, case managers: Establish with the patient their preferred method for reaching relevant services Establish contact with the services or resources identified in the ISP Provide a personalized reference for the patient Explain the case Inform care professionals of past and potential challenges facing the patient Make all necessary external links to appropriate services (community organizations, home care services, specialized clinics, other clinics, and services) Support the patient in navigating the various services provided	Inform the patient and caregiver about available resources Refer to Alzheimer Society Refer patient and caregiver to services available from the local community service centre Communicate with the community pharmacist to facilitate the medication delivery process Call the local community service centre to schedule a short respite stay if necessary Refer to other relevant resources Follow up on referrals

**Engagement:**	**Provide educational self-management support for patients and families:**	**Provide educational self-management support for patients and families:**

	This component is considered an ongoing and transversal process to be performed as needed throughout the intervention. Case managers: Develop a trusting patient-provider relationship in order to positively influence patient’s motivation and engagement in self-care Use motivational interviewing principles and strategies to engage the patient Support the patient to set realistic goals through a “SMART” action plan Support the development of skills related to psychological well-being (anxiety management and assertiveness strategies)	This component aims to maintain patient autonomy for the longest possible time and slow the progression of the AD: Provide education and explanation about AD, the treatment plan, and how to handle behavioural and psychological symptoms of dementia Consider alternatives to driving based on the person’s abilities and reality. Discuss the expected outcomes of the treatment and possible side effects Explain the importance of getting legal and financial affairs in order as soon as possible Provide counselling on medico-legal aspects: power of attorney, protective supervision, etc.

	Support the development of the patient’s ability to monitor, take appropriate actions and know when and how to seek professional help Help the patient prepare for meetings with the various care professionals Ensure the patient is empowered to communicate their goals and to receive the desired care Coach the patient on how to effectively communicate with their relatives: Help the patient establish expectations Help ensure a successful care partnership Provide follow-up/support meetings Organize group self-management support led by lay leaders who suffer from a chronic disease	

	**Develop a relational continuity of care:**	**Develop a relational continuity of care:**

	This is the same resource person who follows the patient and knows their file Serve as the patient’s main contact Advocate for the patient Maintain a relationship of trust (as stated earlier) and enhance the patient’s sense of security Negotiate the services and defend the rights and interests of the patient Adopt a calm, confident, sensitive, friendly, empathic and supportive attitude	Establish a partnership and a relationship of trust with the patient and their caregivers. Provide their direct contact details so the patient or their caregiver can contact the patient navigator if necessary Discuss the diagnosis and the treatment plan

**Success measures**	Case managers are not involved in data collection	Patient navigators collect data including processes and performance indicators.


Key sources (in addition to interviews with experts): MSSS,2019 [52]; MSSS, 2014 [53]; Danish et al., 2020 ([31]; Nicol-Clavet, 2017 [54]. Legend: ADL: activities of daily living; CM: Case manager; CVA: Cerebral vascular accident; ED: Emergency department; EHR: Electronic health record; GP: General practitioner also known as family physician; IADL: instrumental activities of daily living; MSSS: Ministry of Health and Social Services; TIA: Transient ischemic attack.

“The problem with the QAP is that it is Quebec-wide, so there are as many modes of organization as there are different FMGs” one key informant reminded us.

As shown in Table 3, many care coordination activities are common to both programs, yet the difference lies in their components. For instance, patient identification using clinical judgement and relevant clinical assessment tools was found in both programs. However, in the QAP, emphasis was put on cognitive evaluation while in V1SAGES, assessment covered broader risk factors such as the patient’s previous use of health and social care services. Another example is follow-up steps, which were much more detailed and targeted in the QAP than in VISAGES, where elements of this activity remained unspecific since they are dependent on the individualized service plan. Other common activities (but with different components) are: Comprehensive assessment of patient and family needs, goals and patient’s clinical and functional status; co-development of an individualized service plan as a road map for all involved parties; follow-up and change monitoring as well as response to change; communication, referrals and linking patients and caregivers with professionals and services; educational self-management support for patients and caregivers; and relational continuity of care based on trust. Nurses were only involved in data collection in the QAP.

## Discussion

This study provides new information about core care coordination activities that would be relevant to heterogeneous populations of patients with complex needs and could constitute a valuable contribution to facilitating the development of a standard of practice for nurses performing this task. If we aim to develop a capacity building program, the standard of practice constitutes an essential step in the process of designing continuing education.

Moreover, organizational and policy context is widely recognized as an important factor affecting integrated care programs [55]. Another contribution of our study is the illustration, with empirical data, of the interrelation between macro, meso and micro components of integrated care programs. To our knowledge, this is the first study to examine care coordination activities as they are embedded in the design of an integrated care program and to show empirically how the design and policy environment (macro) could have a direct impact on the meso (interprofessional and interorganizational) and micro (nurse, patient and caregiver) components of the program.

### Summary of findings

Our results show several differences and similarities in care coordination activities across these two heterogeneous programs. We also show how these variations are related to the program design which is impacted, in turn, by the policy context. Indeed, one of the main differences between the two programs, related to the policy context, is the scope and financing allocated to the programs. V1SAGES is the initiative of a research team and was developed on a much smaller scale than the QAP, which was a Ministry-driven plan that was extended to the entire province. One of the consequences was that, in the case of the QAP, all nurses working in FMGs kept their caseloads and were trained on early detection of AD in the older people. In parallel, in the V1SAGES and PriCARE program, only a few nurses were trained to carry out case management responsibilities. Wodchis et al. (2020) describe four types of policy support for integrated care, two of which are financing and payment, and workforce and staffing [56]. The authors identify expanding the roles of providers, adding new roles, and finding new ways of working as successful innovative policies, which might explain the positive outcomes of both programs despite the variation in financing.

In addition, while both programs targeted patients with complex health and social care needs, another main difference lies in the characteristics of these needs which had an impact on many design elements and their related care coordination activities. Firstly, the identification of frequent healthcare users was mainly based on administrative data followed by a comprehensive assessment, while in the QAP, patients entered the project following the results of their clinical assessment.

Secondly, the presence of a well-identified medical condition in the QAP target population shaped the patient care trajectory as based on disease management and care while also covering psychosocial needs of patients and their caregivers. Therefore, the design and implementation of the QAP project focused on early detection of AD and early patient referral to specialized care. Clinical tools that were developed and the staff training that were delivered aimed to optimize these two aspects. Consequently, the care coordination activities also focused on early identification assessment, follow-up and referral. The referral process was also facilitated by the presence of well-identified specialized memory clinics. In parallel, frequent users of healthcare services are a more heterogeneous target patient population and present various profiles [57]. A single care trajectory based on disease would not be relevant or feasible. This heterogeneity required a more intensive care coordination intervention such as case management, where less emphasis is placed on disease and more on integrating health and social care services [12]. Interdisciplinary teamwork was reflected in the development of and follow-up on the individualized service plan by health and social care professionals who met regularly and established their communication and information transfer pathways. Nurses’ training also focused on case management theories and case studies.

As for similarities, both programs are committed to a proactive approach where the ultimate goal is the provision of care at the right level—primary care—and time for patients with complex needs. Studies reporting on similar initiatives show better health outcomes and reduction in preventable hospitalizations and emergency departments use [58]. Yet, the implementation and the sustainability of such initiatives have proved challenging over the years [17,59,60]. Key informants from both programs reflected on these challenges in the Quebec context, offering a better understanding of the elements that facilitate and hinder such initiatives and enabling comparisons across countries. Beyond the availability of human resources, these include: 1) the absence of a change champion who is dedicated to achieving organizational change, motivating teams and bringing together stakeholders at the local level. The contributions of operational managers to integrated care programs have been consistently highlighted in the literature [61,62,63], and they have been identified as being instrumental to overcoming active resistance to the changes connected to the innovation’s implementation [64]; 2) the presence of an “*infertile ground*” for collaboration. This means the lack of previous experiences or initiatives related to interorganizational collaboration. In both programs, the degree of interprofessional maturity varied across clinical settings, especially beyond the walls of the FMGs. Collaboration between professionals who belong to different organizations is indeed known to pose a serious challenge given the different management styles and philosophies and the not-easily achieved sense of belonging to a team [65]; 3) the lack of role clarity which clearly constituted an obstacle to the commitment of some family physicians who felt that dementia care does not fall within the primary care level’s range of responsibilities. In their review, Cameron et al (2014) [61] showed the importance for all parties to understand the roles and responsibilities of individuals and agencies or organizations. They also noted the equal importance of having clear legal and financial frameworks for the distribution of these responsibilities; 4) the need to think of the entire continuum of care when designing the program and to make sure it does not create more fragmentation. While the sole purpose of vertical integration is the provision of comprehensive and continuous care across clinical contexts and providers [66], limited resources to develop an integrated care model that covers the entire continuum may lead to this model creating more fragmentation. Appraising the options before pursuing integration would help decision makers to demarcate target groups and establish the most relevant scope of the new service [67]. This strategy is supported by the Laws for Integration as formulated by Leutz, “You can integrate some of the services for all people, or all of the services for some people, but you cannot integrate all the services for all of the people” [68]. And finally, 5) the challenge of ensuring the sustainability of the program when research teams or project managers withdraw and hand it over to local teams. Lessons from previous experiences indicate the necessity of supporting instruments such as care or quality standards, sustained funding mechanisms and regional or local governance structures [69], and the relevance of using collaborative approaches, early in the process, to co-create and implement sustained and successful integrated care models [70].

### Implications of the results on education and practice

In both of the programs included in this study, staff training was delivered prior to the implementation, which confirms the need to invest in the education and training of care coordinators whether through continuing education programs facilitated through the workplace or through the nursing curriculum. Our results identify a set of core activities that would be relevant to heterogeneous target patient populations. Building care coordinators’ capacity to perform these activities seems like a necessity considering the increasing complexity of patients’ needs and situations [71]. In addition to these core activities, competencies such as leadership and interprofessional communication also need to be included in training programs in order to change service delivery towards successful integrated care [72]. Moreover, this study illustrates the embedment of care coordination activities within integrated care programs’ design and emphasizes the need for theoretical aspects of integrated care to be covered too. Indeed, it has been established that transformations towards integrated care require a good understanding of the various dimensions of integration [73].

Our results confirm that the choice of a care coordination program should be primarily based on the needs of the target patient population [18]. They also show how patients’ complex needs shape the design and implementation of the program. Indeed, our findings confirm that integrated care pathways, despite their proven benefits [74], are more effective when the care trajectories are predictable [75] and patients’ complex needs are homogeneous, and may be less effective in variable patient trajectories—where care needs to be more flexible [76]—such as those of frequent users of healthcare services. Moreover, the more complex the patient’s needs, the more the care trajectories put strain on them due to the multiple options and multiple alternatives they need to consider [75]. Our results suggest that, for these populations, a case management model of care coordination is perhaps the most relevant solution to implement given the intensity of its approach [12]. Case managers would play a crucial role in modelling patients’ care trajectories according to their complex and evolving needs.

### Strengths and limitations

Care coordination and integrated care programs are complex, inherently context sensitive, and evolving over time [77]. Our comparative case study has the strength of illustrating these three components through two very different cases and confirming that this remains true despite the heterogeneity of programs. Another strength would be the use of The Integrated Care Case Study Descriptive Template. This conceptual framework allows us to structure data collection and analysis and include core components of the two innovations, but also to share knowledge across these examples that may be compared to other contexts. Finally, the input provided by key informants who had an in-depth knowledge of the programs, which gives considerable weight to our results.

As for limitations, data collection could have included observations or interviews with case managers and care navigators. However, the pandemic impeded their availability. Also, we did not examine patients’ experiences with care coordination but we plan to do so as part of a subsequent phase.

## Conclusion

Comparative case studies of integrated care offer an in-depth understanding of how programs are designed and implemented worldwide to reduce fragmentation and improve the patient care experience. While they are intended to provide insights with regard to challenges in particular contexts, they can facilitate learning across borders and build strong national knowledge. Overall, organizational context, program design, policy environment, as well as the characteristics of patients’ complex needs have a major impact on implementation and delivery of care coordination programs. Despite the differences across care coordination programs targeting patients with complex needs, they share a commonality in that they tend to be adjusted to the homogeneity or heterogeneity of needs. As the complexity of patients’ needs grows, so does the necessity and urgency to build the care coordinators’ capacity for integrated care. Education and training programs should include clinical, leadership and professional competencies, as well as theoretical aspects of integrated care. Both the higher education institutions and workplaces are responsible for this capacity building.

## Additional File

The additional file for this article can be found as follows:

10.5334/ijic.6729.s1 Appendix A. The document review.
