# Supplementary material for: Nursing Care Coordination in Primary Healthcare for Patients with Complex Needs: A Comparative Case Study
Source: Int J Integr Care. 2023 Feb 2;23(1):5. doi: 10.5334/ijic.6729 (PMC9912854; doi:10.5334/ijic.6729)
Supplement: Appendix A. — The document review. [file ijic-23-1-6729-s1.pdf]

**Appendix A: The document review**

Following Merriam's (2009) case study methodological approach to document review, four documentary sources were searched and analyzed. Below is a non-exhaustive list of documents of each source type:

1) **Public records:**

- Bocti C, Bergman H, Azuelos E, Brodeur C, Kirk J, Lacombe G, Massoud F, Patry C, Thorn K. (2014). Care protocol. Interdisciplinary clinical process in primary care. Alzheimer's disease and related disorders. [Protocole de soins. Processus de clinique interdisciplinaire en première ligne. Maladie Alzheimer et les maladies apparentées]. Available from : https://www.mcgill.ca/familymed/files/familymed/rd_protocole_de_soins_alzheimer_1e_ligne-20150127.pdf
- Ministry of Health and Social Services. (2019). Practical guide for registered nurses working in a family medicine group or an academic family medicine group. From an interprofessional collaboration perspective. *[Guide pratique à l'intention des infirmières cliniciennes qui travaillent dans un groupe de médecine de famille ou un groupe de médecine de famille universitaire. Dans une perspective de collaboration interprofessionnelle]*. Available from: https://publications.msss.gouv.qc.ca/msss/document-002376/
- Ministry of Health and Social Services. (2018). Alzheimer's disease and major neurocognitive disorders. Training and coaching. *[Alzheimer et autres troubles neurocognitifs majeurs. Formation et mentorat]*. https://www.msss.gouv.qc.ca/professionnels/maladies-chroniques/alzheimer-et-autres-troubles-neurocognitifs-majeurs/formation-et-mentorat/
- Ministry of Health and Social Services. (2017). Implementation guide for the deployment of the best clinical and organizational practices in IHSSCs and IUHSSCs. Second phase 2016 to 2019. *[Guide de mise en œuvre pour le déploiement des meilleures pratiques cliniques et organisationnelles dans les CISSS et les CIUSSS. Seconde phase des travaux 2016 à 2019]*. Available from : https://publications.msss.gouv.qc.ca/msss/document-001825/
- Ministry of Health and Social Services. (2015). Primary care interdisciplinary clinical process. *[Processus clinique interdisciplinaire en première ligne]*. Available from: https://publications.msss.gouv.qc.ca/msss/document-001071/#:~:text=Le%20Processus%20clinique%20interdisciplinaire%20en,dont%20la%20maladie%20d'Alzheimer.&text=Cette%20publication%20est%20disponible%20en%20version%20%C3%A9lectronique%20seulement.
- Ministry of Health and Social Services. (2014). General project framework - Targeted implementation projects in FMGs to improve access to health and social services with the support of secondary and tertiary care levels. *[Cadre général de projet - Projets d'implantation ciblée en GMF pour rehausser l'accès aux services de santé et services sociaux avec le soutien des 2e et 3e lignes de services]*. Available from: https://publications.msss.gouv.qc.ca/msss/document-001090/
- Ministry of Health and Social Services. (2014). Book of structural and content requirements - Targeted implementation projects in FMGs to improve access to health and social services with the support of secondary and tertiary care levels. *[Cahier des exigences structurelles et de contenu. Projets d'implantation ciblée en GMF pour rehausser l'accès aux services de santé et*

*services sociaux avec le soutien des 2e et 3e lignes de services]*. Available from : https://publications.msss.gouv.qc.ca/msss/document-001091/

- Vedel I, Couturier Y. (2016). Results of evaluative research and course of action for extending the "Ministerial initiative on Alzheimer's disease and other major neurocognitive disorders across Quebec" *[Résultats de la recherche évaluative et pistes d'action pour la généralisation à l'ensemble du territoire de « l'Initiative ministérielle sur la maladie d'Alzheimer et autres troubles neurocognitifs majeurs »*. Available from : https://www.mcgill.ca/familymed/files/familymed/final_report_for_msss_20161024_execsummary_eng.pdf
- …

2) **Private documents**: (protocols and standards of care, care coordinators' training records, clinical tools, communication and coordination tools, and patients' informed consent forms):
   - Godbout J, Chouinard MC, Warren M, Danish A. (2019). *Case management training.*
   - Information and consent form of the user to the communication of personal information (V1SAGES).
   - User's consent form to the individualized service plan (ISP) or the interdisciplinary intervention plan (IIP) (V1SAGES).
   - Professionals' invitation form to a meeting to develop an individualized service plan (ISP) for/with the user (V1SAGES).
   - Individualized service plan form OR interdisciplinary intervention plan form.
   - Initial comprehensive assessment form
   - Standard of care for case management.
   - …

3) **Unpublished records**: (Master's theses)
   - Guillette, M. (2016). *From politics to changes in practice: translating the Quebec Alzheimer Plan in its local forms*. *[De la politique aux changements de pratiques : la traduction du Plan Alzheimer du Québec dans ses formes locales]*. [Master's thesis]. Sherbooke (Quebec). Available from: https://savoirs.usherbrooke.ca/bitstream/handle/11143/9734/Guillette_Maxime_MServSoc_2016.pdf?sequence=5&isAllowed=y
   - Nicol- Clavet, N. (2017). Family medicine group nursing practice within the framework of the Quebec Alzheimer's Plan. *[La pratique infirmière en groupe de médecine de famille dans le cadre du Plan Alzheimer Québec]*. [Master's thesis]. Sherbooke (Quebec). Available from: https://savoirs.usherbrooke.ca/bitstream/handle/11143/11567/Nicol_Clavet_Noemie_MA_2017.pdf?sequence=3&isAllowed=y

4) **Research publications**: Primary and secondary analysis of published articles on the programs (please see the references list).
